# Interactions of Osteoprogenitor Cells with a Novel Zirconia Implant Surface

**DOI:** 10.3390/jfb11030050

**Published:** 2020-07-16

**Authors:** Thomas Munro, Catherine M. Miller, Elsa Antunes, Dileep Sharma

**Affiliations:** 1 College of Medicine and Dentistry, James Cook University, 14-88 McGregor Road, Smithfield 4878, QLD, Australia; thomas.munro@my.jcu.edu.au; 2 College of Public Health, Medical and Veterinary Sciences, James Cook University, 14-88 McGregor Road, Smithfield 4878, QLD, Australia; kate.miller1@jcu.edu.au; 3 The Australian Institute of Tropical Health and Medicine (AITHM) James Cook University, 14-88 McGregor Road, Smithfield 4878, QLD, Australia; 4 College of Science & Engineering, James Cook University, 1 James Cook Drive, Douglas, Townsville 4814, QLD, Australia; elsa.antunes1@jcu.edu.au

**Keywords:** dental implants, Yttria-tetragonal zirconia, titanium, osseointegration, surface properties, immunocytochemistry, cell attachment, cell migration

## Abstract

*Background:* This study compared the in vitro response of a mouse pre-osteoblast cell line on a novel sandblasted zirconia surface with that of titanium. *Material and Methods:* The MC3T3-E1 subclone 4 osteoblast precursor cell line was cultured on either sandblasted titanium (SBCpTi) or sandblasted zirconia (SBY-TZP). The surface topography was analysed by three-dimensional laser microscopy and scanning electron microscope. The wettability of the discs was also assessed. The cellular response was quantified by assessing the morphology (day 1), proliferation (day 1, 3, 5, 7, 9), viability (day 1, 9), and migration (0, 6, 24 h) assays. *Results:* The sandblasting surface treatment in both titanium and zirconia increased the surface roughness by rendering a defined surface topography with titanium showing more apparent nano-topography. The wettability of the two surfaces showed no significant difference. The zirconia surface resulted in improved cellular spreading and a significantly increased rate of migration compared to titanium. However, the cellular proliferation and viability noted in our experiments were not significantly different on the zirconia and titanium surfaces. *Conclusions:* The novel, roughened zirconia surface elicited cellular responses comparable to, or exceeding that, of titanium. Therefore, this novel zirconia surface may be an acceptable substitute for titanium as a dental implant material.

## 1. Introduction

The loss of teeth or edentulism is a debilitating condition affecting approximately 15.5% of the Australian population in its severe form, resulting in fewer than 21 teeth in adult dentition [1]. Edentulism can directly result in physical impairment, functional limitation, psychological disability, and social disability, along with handicap [2]. The successful management of partial and full mouth edentulism involves the placement of dental implants into the jaw bone, which provides anchorage and support for the fixed artificial tooth/teeth (prosthesis).

Modern implant dentistry began in the 1950s when Per-Ingvar Brånemark, a Swedish professor, stumbled upon a phenomenon he called “osseointegration” [3]. The success of endosseous implants is directly related to osseointegration: a process of implant–bone interaction that ultimately leads to bone-to-implant anchorage, which is crucial for the long-term success of the implant [4]. The first patient was successfully treated in 1965, using a titanium screw implant [3]. Since then, millions of patients worldwide have been treated with dental implants, with titanium having established itself as the preferred material [3,5,6]. 

Titanium is known to possess excellent mechanical strength and is highly biocompatible, whereby the formation of an oxide layer facilitates cellular interaction and osseointegration. This biocompatible material historically enjoys high success rates, making it the most widely used material for osseous implants today [3,7,8,9]. Despite being the gold standard, titanium has its own drawbacks. Its grey hue can be a significant aesthetic issue, and there can be a corrosion of metal that can trigger a hypersensitivity reaction or lead to an accumulation of titanium within internal organs [3,5]. Furthermore, a shift in patient preference towards a non-metallic solution has resulted in a demand for alternative implant systems, ushering in the advent of ceramic dental implants [3,5,6,10,11,12,13,14,15].

Ceramic materials are commonly used in dentistry due to a high biocompatibility and excellent aesthetics, mimicking the appearance of a natural tooth [5]. Ceramics have been used for various applications such as the fabrication of crowns and bridges, orthodontic brackets, and implant abutments [16]. Recently, Yttria-tetragonal zirconia polycrystal (Y-TZP) has been proposed as an alternative material for implants as it is tooth-coloured, resistant to plaque formation, and biocompatible with suitable mechanical properties. However, there is a need to evaluate its properties further [3,4,6,16,17,18,19,20,21,22].

The micro- and nano-structure of implant surfaces is a significant factor for titanium and zirconia to achieve successful and reliable osseointegration [5,23,24,25,26,27,28,29]. Hence, various surface modifications have been used to modulate the physical and chemical properties aiming to improve bone-to-implant interaction [5,17,25,29]. At the molecular level, modified implant surfaces can increase the adsorption of serum proteins, cytokines, and mineral ions and better retain a fibrin clot, subsequently promoting cellular migration and attachment [30,31,32]. Different implant surface treatments and materials will possess unique characteristics that can affect the host cellular response as shown by in vitro studies using several cell lines including human fetal osteoblasts, human mesenchymal stem cells, and mouse calvaria cells MC3T3-E1 [17,31,33,34,35,36,37,38,39,40]. Hence, cell culture assays are pivotal to understanding the cell response to any new implant material surface [17,33]. The unique surface characteristics of implants may be obtained through various methods of machining, blasting, acid-etching, coating, laser technology, or a combination of procedures. As with titanium, in vitro and in vivo studies have confirmed that zirconia-based ceramic surfaces are chemically inert with minimal local or systemic adverse responses [41]. 

**Figure 1:**
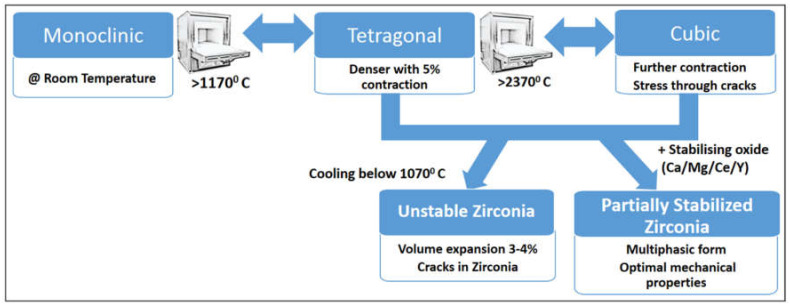
The three crystalline phases of zirconia. The desirable mechanical properties of zirconia are possessed by a state of tetragonal and cubic forms, stabilised below 1070 °C to retain these properties with the addition of alumina, magnesia, cerium, and/or yttria.

Creating an optimised zirconia topography without compromising the biomechanical stability is a technical challenge that, so far, has resulted in increased failure rates under function with numerous zirconia implant fractures [18,19,20,42,43,44]. Zirconia’s physical characteristics and mechanical properties are a significant impedance in its development for implant fabrication. Of the three crystalline forms in which zirconia exists (monoclinic, tetragonal, and cubic), the desirable mechanical properties are achieved with the advent of partially stabilised zirconia. However, manufacturing processes induce an inherent stress and cracks within the material and render it unstable (Figure 1). The addition of various stabilising agents such as magnesia, cerium, or yttria at different concentrations and combinations has yielded various forms of partially stabilised zirconia with a significant enhancement in structural strength due to enhanced resistance to slow crack growth [45]. Currently, yttria partially stabilised zirconia is commonly employed for dental implants that are currently available for clinical use [46,47]. Amended manufacturing processes have aimed to produce a micro-roughened zirconia implant with decreased fracture rates and improved fatigue strength enhancing the clinical performance of zirconia implants [18]. However, the optimal design for zirconia implant osseointegration is yet to be determined [24,48,49,50,51]. Zirconia implants have shown superior soft-tissue responses, biocompatibility, and aesthetics with comparable osseointegration to titanium; however, additional research is required further improve zirconia dental implants and validate them as a viable alternative to the titanium implant [16,23]. Therefore, the rationale of this study was to characterise a novel zirconia implant surface and evaluate its osseointegration potential compared to a titanium surface, using in vitro cell culture assays focused on cellular viability and proliferation, attachment, cytoskeletal changes, and migration.

## 2. Materials and Methods

All the cellular assays were designed and conducted in compliance with the Minimum Information About a Cellular Assay (MIACA) guidelines [52,53]. Modified Consolidated Standards of Reporting Trials (CONSORT) guidelines for preclinical in vitro studies on the dental materials checklist was utilised to report our findings [54]. Ethics approval was not required for this in vitro study.

### 2.1. Sample Preparation

Commercially procured titanium alloy (CpTi) was used to fabricate discs that were 14 mm in diameter and 3.5 mm in thickness. The yttria-tetragonal zirconia polycrystal (Y-TZP) used was obtained by sintering commercial 3 mol% yttria partially stabilised zirconia powder (30% monoclinic and 70% tetragonal) to produce discs that were 16 mm in diameter and 3 mm in thickness. A ready-to-press powder was uniaxially pressed at 3000 kgf/cm^2^ pressure in a pellet press die followed by sintering at 1450 °C for 2 h with a constant heating rate of 10 °C/min. The sintered discs were characterised by 100% tetragonal crystalline structure with a bulk density of 6.07 g/cm^3^. After sintering, all samples were wet ground on silicon carbide abrasive paper and polished to obtain a smooth surface. Prior to surface treatment, the samples were ultrasonically cleaned in a 100% ethanol bath for 15 min and then in distilled water for 10 min to remove any surface contamination or debris from the polishing process.

Sandblasting is a commonly used subtractive surface treatment with several sandblasted ceramic dental implants commercially available; however, the exact details of these commercial products are limited [3,6,16,44,55,56,57,58,59]. In this study, both the CpTi and Y-TZP discs were surface treated by sandblasting with alumina (Al_2_O_3_; grain size 150–200 μm; Korox 250, BEGO GmbH & Co., Bremen, Germany) at 0.6 MPa pressure for 3 min at a distance of 6 cm in a BEGO Duostar Z2 (BEGO GmbH & Co., Bremen, Germany), creating a sandblasted yttria-tetragonal zirconia polycrystal surface (SBY-TZP) and a sandblasted commercially pure titanium surface (SBCpTi). Before use in cell culture experiments, all specimens were ultrasonically cleaned with purified water (Milli-Q^®^ system, Merck Millipore Corporation, Darmstadt, Germany) for 15 min, followed by autoclave sterilisation at 134 °C for 20 min.

### 2.2. Surface Characterisation

After sterilisation, discs of CpTi and Y-TZP were used for surface analysis using laser scanning microscopy, scanning electron microscopy (SEM), and contact angle measurement, comparing untreated and sandblasted surfaces to elucidate the effect of sandblasting on surface characteristics.

#### 2.2.1. Laser Scanning Microscopy

To assess the surface topography of the discs, images were acquired with a laser scanning microscope (LEXT OLS4100, Olympus Corporation, Tokyo, Japan). Three discs of each sample type (untreated Y-TZP and CpTi; sandblasted Y-TZP and CpTi) were used to obtain a range of measurements at three randomly selected sites on each disc. The following measurements were recorded with a Gaussian filter to separate the roughness from errors of form or waviness; discs were characterised by height, spatial, and hybrid parameters as described by Wennerberg and Albrektsson [30].

- Sa (μm): arithmetical mean height; average height deviation (above and below) from the mean plane height of the surface. A measure of surface roughness.
- Sdr (units): developed interfacial area ratio; the ratio of the additional surface area created by the texture compared to the planar area.
- Ssk (units): skewness; asperity of the surface.
- Sku (units): kurtosis; a measure of the sharpness of the roughness topography.
- Str (units): texture aspect ratio; determines if the surface topography is uniform or irregular.

The resulting surface topography from sandblasting was also represented by three dimensional-topography models [30,60].

#### 2.2.2. Scanning Electron Microscopy

Topographical disc analysis was also determined with SEM analysis (Phenom™ G2 pro, Phenom-World BV, Eindhoven, The Netherlands). Following gold sputtering (Spi-Module™ Sputter Coater, SPI Supplies, West Chester, PA, USA), three discs of each sample type (sandblasted and untreated CpTi; sandblasted and untreated Y-TZP) were used to obtain images at three randomly selected sites on each disc.

#### 2.2.3. Contact Angle Measurement

The sessile drop method was used for contact angle measurements whereby 80 μL of purified water was deposited onto both the untreated and sandblasted dry disc (CpTi and Y-TZP) surfaces at room temperature. Three discs of each sample were used to obtain images. Images were calibrated to the scale in each image, and the angles were measured using ImageJ software (version 1.53a, National Institute of Health, Bethesda, NY, USA) that was repeated three times on each side of the sessile drop.

A relationship between surface roughness and wettability is shown by Wenzel’s equation r_a_(γ_sv_−γ_sl_) = γ_lv_cosθ_w_, whereby *r*_a_ is the roughness factor and θ_w_ is the contact angle of a rough surface. The equation shows that if the roughness factor increases, then cosθ_w_ will increase, resulting in a decreased resultant contact angle [61].

### 2.3. Cell Culture

Mouse calvaria cells MC3T3-E1 subclone 4 (CRL-2593 ATCC, Manassas, VA, USA), an osteoblast precursor cell line, was utilised in this study. MC3T3-E1 cells were expanded in culture flasks with complete media (CM) consisting of Dulbecco’s modified Eagle medium (DMEM), supplemented with 10% fetal bovine serum (FBS) and 1% penicillin–streptomycin solution (Sigma-Aldrich, Castle Hill, NSW, Australia) incubated at 37 °C, 5% CO_2_, and 90% humidity. Media was refreshed every 3 days, and the cells were passaged at 95% confluence as confirmed using conventional microscopy (Nikon Eclipse TS100, Nikon Instruments, Tokyo, Japan). For experiments assessing cellular response, cells were lifted with trypsin (Sigma-Aldrich, Castle Hill, NSW, Australia) and seeded onto the SBY-TZP and SBCpTi discs only.

#### 2.3.1. Cell Morphology

The cytoskeletal arrangement of the MC3T3-E1 cells was examined by an inverted epi-fluorescence microscope (Olympus IX53 epifluorescence microscope, Olympus Corporation, Tokyo, Japan) to visualise the cytoskeletal protein, actin, which was stained with 2% Flash Phalloidin™ red solution (BioLegend, San Diego, CA, USA) according to the manufacturer’s instructions. Initially, 1.6 mL of media containing MC3T3-E1 cells at 1×10^6^ cells/mL was used to seed cells onto the SBY-TZP (n = 4) and SBCpTi (n = 4) discs over 24 h. Subsequently, cells were fixed for 10 min with 4% paraformaldehyde at room temperature and permeabilised using 0.5% Triton X-100 (Sigma-Aldrich, Castle Hill, NSW, Australia). FBS (5%) was used as a blocking agent prior to incubation with Flash Phalloidin™ red solution for 20 min at room temperature. Then, stained cells were imaged and analysed to determine the percentage of Flash Phalloidin™ red solution fluorescence compared to the background (disc) as a percentage to quantify the amount of fluorescence of MC3T3-E1 cells on SBY-TZP and SBCpTi discs [62,63,64].

The cytoskeletal arrangement of the cells was also examined under SEM using a protocol adapted from Fischer et al. [65]. First, 1.6 mL of media containing cells at 1 × 10^6^ cells/mL was seeded onto SBY-TZP (n = 4) and SBCpTi (n = 4) discs and incubated for 24 h. Attached cells were fixed with 3% glutaraldehyde; then, they were dehydrated in graded concentrations of ethanol (25%, 50%, 75%, 95%, 100%) for 5 min at each concentration. Subsequently, discs were placed in a 1:1 solution of hexamethyldisilazane (HMDS) and ethanol for 15 min, followed by 100% HMDS for 5 min. Samples were dried for 4 h within a fume hood before gold sputter coating and SEM evaluation.

#### 2.3.2. Cell Viability and Cell-Covered Area

To evaluate the viability of the cells on the SBY-TZP and SBCpTi discs, live and dead cells were analysed at days 1 and 9. A Cytopainter Cell Plasma Membrane Staining Kit at 20% (ab219941 Abcam, Melbourne, Australia) was used to stain live cells, and 2% propidium iodide stain (ThermoFisher, Scoresby, Australia) was used to counterstain dead cells. First, 1.6 mL of media containing cells at 1 × 10^6^ cells/mL was seeded onto the SBY-TZP (n = 4) and SBCpTi (n = 4) discs. The stain solution was added to each well and incubated for 20 min in the dark. Images were obtained using an inverted epifluorescence microscope and analysed using ImageJ software to determine a live–dead ratio and cell-covered area [62,63,64].

#### 2.3.3. Cell Proliferation

The MC3T3-E1 cells were seeded onto the SBY-TZP (n = 8) and SBCpTi (n = 8) discs, as well as eight cultures wells (positive control). First, 1.6 mL of media containing cells at 1× 10^6^ cells/mL was used to seed cells onto each disc or well. The assay was run with 10% *v/v* Resazurin (Sigma-Aldrich, Castle Hill, NSW, Australia) to determine cellular proliferation at 1, 3, 5, 7, and 9 days. Resazurin solution was added to the wells at each time point and incubated for 5 h in the incubator. Media from each specimen was transferred to a 96-well plate (in triplicate: 3 wells of 100 μL each), and the absorbance of resorufin (reduction product of resazurin) at 570 nm and 600 nm wavelength was recorded using a microplate absorbance reader (iMark™ Microplate Absorbance Reader, BioRad Laboratories, Hercules, CA, USA). The percentage of resorufin was calculated using the values obtained for stock solution (without cells). The raw data were transformed as a factor of surface area, with the SBY-TZP dimensions (16 mm diameter) being greater than the SBCpTi discs (14 mm).

#### 2.3.4. Cell Migration

Migration was assessed using a scratch-healing assay whereby cells at 1 × 10^6^ cells/mL were seeded on the SBY-TZP (n = 4) and SBCpTi (n = 4) discs (1.6 mL of media) and cells were grown until confluent. Two scratches across each disc were made using a sterile 200 μL pipette tip and followed by thorough washing with phosphate-buffered saline (PBS; Sigma-Aldrich, Castle Hill, NSW, Australia) to remove detached cells. The scratches were imaged using an inverted epifluorescence microscope after 0, 6, and 24 h of incubation with Flash Phalloidin™ Red solution as per the manufacturer’s instructions. The images were analysed, with the result represented as a percentage of the initial open area of the scratch covered by cells at each time point using ImageJ software [66].

### 2.4. Statistical Analysis

IBM SPSS Statistics 20 (IBM SPSS Inc., Chicago, IL, USA) were used for statistical analysis. The Mann–Whitney U-test and ANOVA was used to make comparison among the groups; results are presented as median ± interquartile range and mean ± standard deviation, respectively. A *p*-value < 0.05 was considered statistically significant.

## 3. Results

### 3.1. Surface Characterisation

#### 3.1.1. Sandblasting-Affected Surface Topography in a Unique Manner for the Y-TZP and CpTi Discs

**Figure 2:**
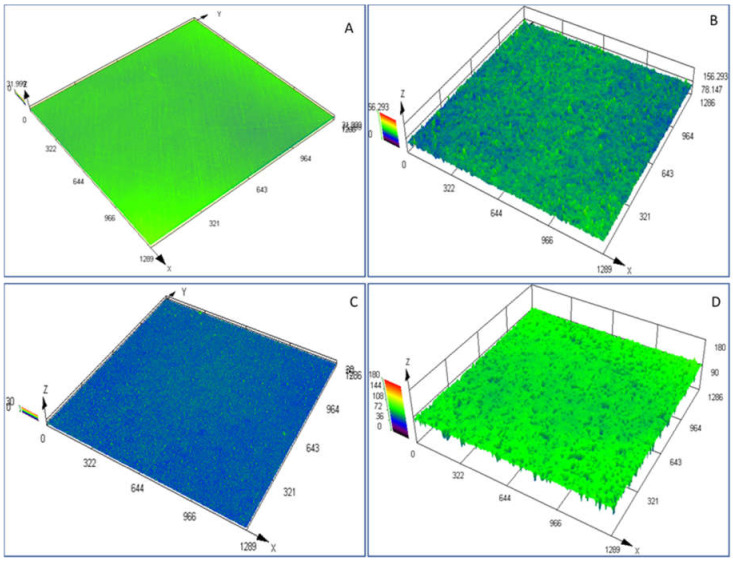
Three-dimensional laser microscopy images of untreated and sandblasted Y-TZP and CpTi. Images were obtained on three discs at three randomly selected sites using digital laser scanning microscopy, and representative wireframes were generated. Wireframes are shown in micrometres (µm). A and B, representative images (10× magnification) of untreated CpTi (**A**) and SBCpTi (**B**). C and D, representative images (10× magnification) of untreated Y-TZP (**C**) and SBY-TZP (**D**).

The untreated Y-TZP and CpTi surfaces revealed a smooth topography, with limited signs of texture that resulted from the manufacturing processes (Figure 2). The untreated Y-TZP and CpTi surfaces had no significant difference in Sa, Sdr, or Sku value, meaning that both surfaces were of similar sharp roughness and surface area (Table 1). The two surfaces were significantly different in Ssk (*p* = 0.0076) and Str (*p* = 0.001), confirming that the untreated Y-TZP surface had a significantly more uniform topography and its texture was skewed above the mean surface plane compared to CpTi, which had relatively equal height distribution above and below the mean plane (Table 1).

Both Y-TZP and CpTi had a significant increase in the surface parameter Sa with the sandblasting process compared with the untreated surfaces (for both CpTi and Y-TZP; *p* = 0.008. Table 1). The other parameters of Ssk, Sku, and Str show that both treated surfaces had a height distribution skewed below the mean plane, which was spiked in texture and spatially isotropic (Table 1). This was evident in the three-dimensional surface models obtained from laser scanning microscopy on both the SBY-TZP and SBCpTi samples (Figure 2B,D). SBY-TZP had a significant increase in Sa (*p* = 0.008), Sdr (0.007), Ssk (*p* = 0.0022), and Str (*p* = 0.0022) compared with untreated Y-TZP, showing a surface that had increased in roughness, surface area, and a texture skewed below the mean plane, describing a non-uniform surface topography. SBCpTi had a significant increase in Sa (*p* = 0.008), Sdr (0.002), and Sku (*p* = 0.001) compared to untreated CpTi, revealing an increased roughness, surface area, and sharp surface topography (Table 1; Figure 2).

The Sa values were comparable for SBY-TZP and SBCpTi, with no statistically significant difference (*p* = 0.6320); therefore, the surfaces were of similar surface roughness. Titanium showed a sharper surface (higher Sku value; not significant) that was more uniform in texture (lower Str value; not significant) and had a height distribution closer to the mean surface plane (lower Ssk value; not significant) compared to zirconia (Table 1). Although the surface differences were visually apparent (Figure 2), the parameters did not reach statistical significance. SBY-TZP had a height distribution further below the mean plane of the surface (Figure 2), where 10× magnification shows a heavily pitted surface with limited surface features evident between pits. Table 1 and Figure 2 show that the morphology of the SBCpTi surface revealed a more prominent surface topography with a significant difference in Sdr (*p* = 0.001) compared to the SBY-TZP surface, showing a significant difference in the surface area of the sandblasted surfaces; titanium had a more developed nano-topography. In summary, the same sandblasting procedure resulted in a Y-TZP surface with distinct topographical properties compared to CpTi.

**Figure 3:**
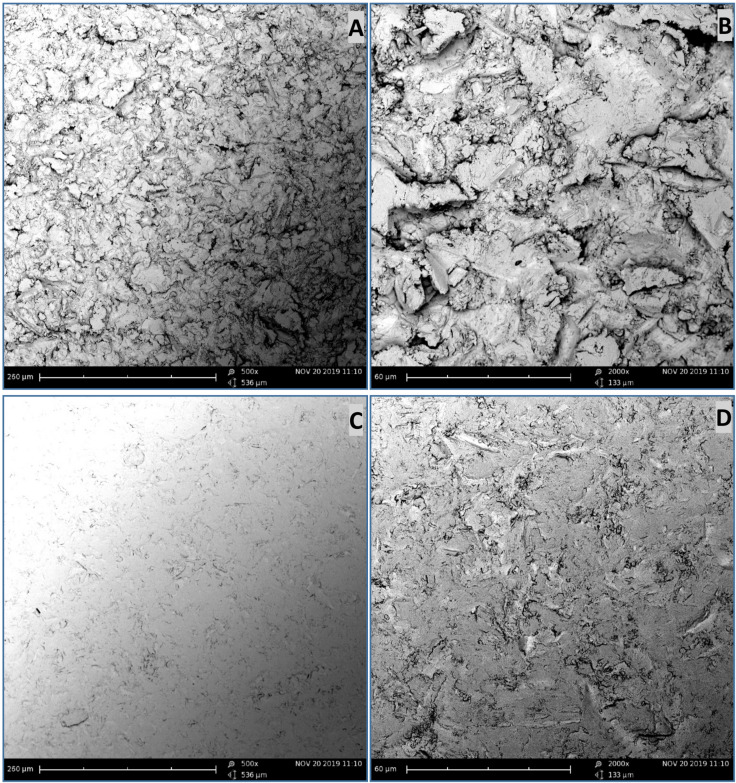
SEM micrographs of the CpTi and Y-TZP surfaces before cell culturing. Topographical images were obtained (500× and 2000× magnification) with an SEM, taken on three discs each of SBCpTi and SBY-TZP at three randomly selected sites following gold sputtering. Representative images of the sandblasted surfaces were chosen: SBCpTi 500× (**A**) and 2000× (**B**); SBY-TZP 500× (**C**) and 2000× (**D**).

Representative SEM micrographs of the SBCpTi and SBY-TZP discs are shown in Figure 3. Sandblasted surfaces were free from residual particles following the cleaning process, confirming no surface contamination by the Al_2_O_3_ particles used for the sandblasting treatment. Sandblasted CpTi was shown to have a consistently complex surface topography without a discernible pattern. Sandblasted Y-TZP showed almost untreated areas of the surface interspersed with irregular surface features, which occur without any visible pattern. SEM micrograph showed that whilst the two surfaces have received the same surface treatments, the resultant topography is unique for both titanium and zirconia.

#### 3.1.2. Sandblasting Improved the Wettability of both Y-TZP and CpTi Discs

**Figure 4:**
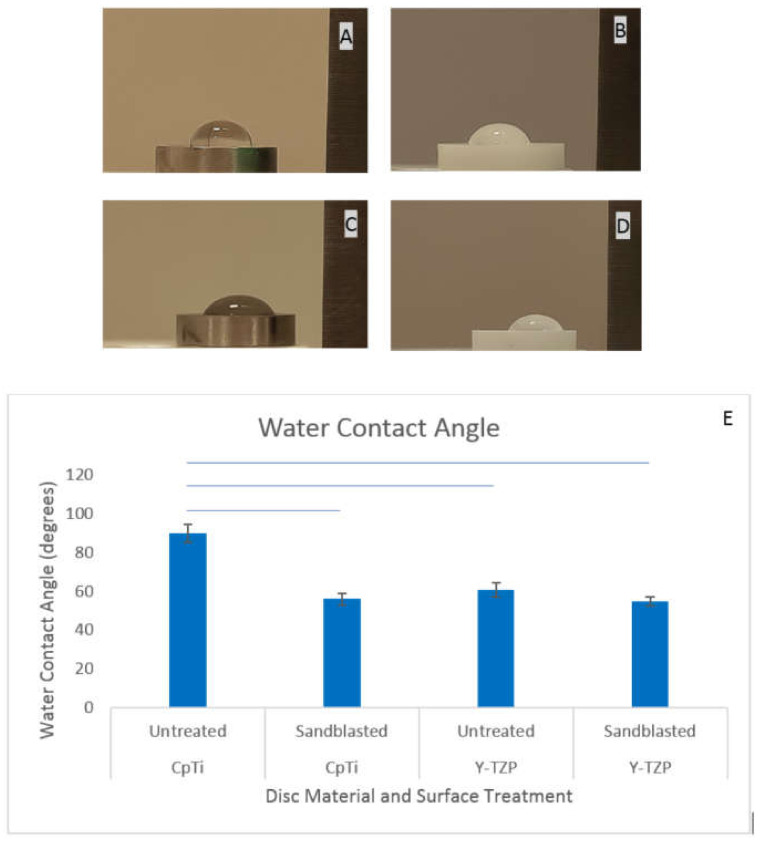
Water contact angle measurements of the CpTi and Y-TZP surfaces. Contact angles were assessed with the sessile drop method on the untreated and sandblasted CpTi and Y-TZP dry disc surfaces at room temperature. Representative images of untreated and sandblasted zirconia and titanium were chosen: untreated CpTi (**A**); untreated Y-TZP (**B**); SBCpTi (**C**); and SBY-TZP (**D**). Images were analysed using ImageJ software (**E**). Results are presented as mean ± standard deviation (n = 3). Lines within the graph show which samples had a statistically significant difference (*p* < 0.05).

The wettability of the untreated surfaces was significantly different to the wettability of treated surfaces (*p* = 0.0022) with sandblasting treatment on both titanium and zirconia discs resulting in a reduction in contact angle (Figure 4). As noted in Table 1 and Figure 2, the surface roughness of the SBCpTi and SBY-TZP increased, and as such, the contact angle decreased. Roughened titanium was seen to have a significant reduction in contact angle compared to the untreated titanium surface: approximately a 34-degree reduction (*p* = 0.002). However, no significant difference was noted between the two sandblasted surfaces (SBCpTi and SBY-TZP) with contact angles of approximately 56 degrees and 55 degrees, respectively (*p* > 0.05).

### 3.2. Cellular Responses of MC3T3-E1 Cells to Sandblasted Surfaces of Y-TZP and CpTi Discs

Since the initial cell response is known to be modulated by the altered surface topography and chemical properties of the implant material, SBCpTi and SBY-TZP were the only disc surfaces tested [17,31,33,34,35,36,37,38,39,40].

#### 3.2.1. Cells Showed Morphological Differences between Sandblasted Y-TZP and CpTi Discs

**Figure 5:**
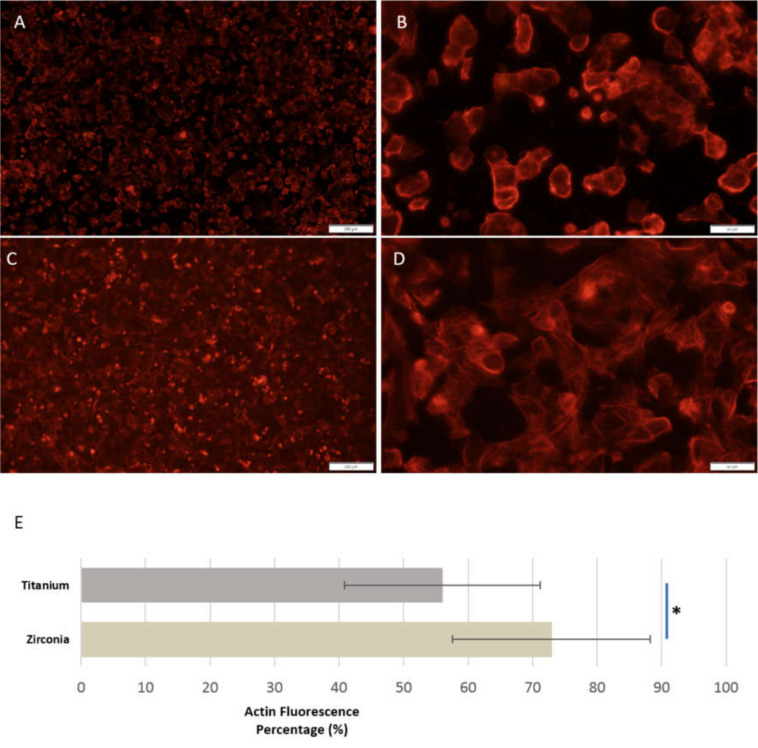
The cytoskeletal arrangement of the MC3T3-E1 cells across SBCpTi and SBY-TZP surfaces. MC3T3 cells were seeded on to SBY-TZP (n = 4) and SBCpTi (n = 4) discs and incubated for 24 h. Cells were imaged on each sample using Flash Phalloidin^TM^ Red solution and an epifluorescence microscopy. A and B are representative images of cells across SBCpTi; 10× magnification (**A**), 40× magnification (**B**). C and D are representative images of cells across SBY-TZP; 10× magnification (**C**), 40× magnification (**D**). The amount of fluorescence was measured to obtain a percentage area of cytoskeletal arrangement using ImageJ software. Results are presented as median ± interquartile range (**E**). * denotes a significant difference in the percentage of actin fluorescence compared to total area on SBY-TZP discs compared with SBCpTi discs (*p* < 0.05).

**Figure 6:**
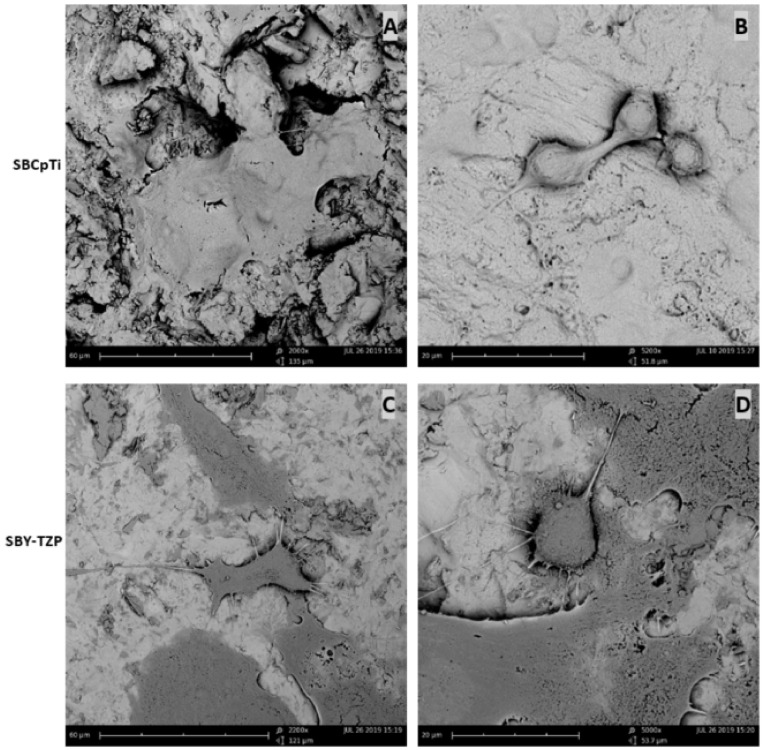
SEM micrographs of cell morphology on SBCpTi and SBY-TZP surfaces. Cell morphology was imaged by SEM, taken on four discs each of SBCpTi and SBY-TZP after being desiccated to critical point and gold sputter coating. Representative images of the sandblasted surfaces were chosen: SBCpTi 2000× (**A**) and 5200× (**B**), SBY-TZP 2200× (**C**), and 5000× (**D**).

After 24 h of incubation, cells were found to be adhering onto both SBCpTi and SBY-TZP discs (Figure 5). Flash Phalloidin™ staining showed that the extent of early cellular spread was denser on zirconia with a marked difference in the number of cell-to-cell contacts when compared to titanium. Images from titanium discs revealed a prominent number of spherical cells with limited extensions and cell-to-cell contact. MC3T3-E1 cells cultured on the zirconia surface showed well-organised actin fibres and filopodiae seeking intercellular contact. This was supported by the percentage of actin fluorescence being significantly higher on SBY-TZP compared with SBCpTi (*p* = 0.0015). SEM confirmed the findings of the adhesion assay, showing SBCpTi to have limited sites of cell-to-cell contacts with limited cellular extensions (Figure 6C,D) in contrast to SBY-TZP, wherein the extent of cellular spread was greater with a marked increase in intercellular contacts (Figure 6A,B).

#### 3.2.2. Sandblasted Y-TZP and CpTi Discs Showed Comparable Proliferation and Viability of MC3T3-E1 Cells

**Figure 7:**
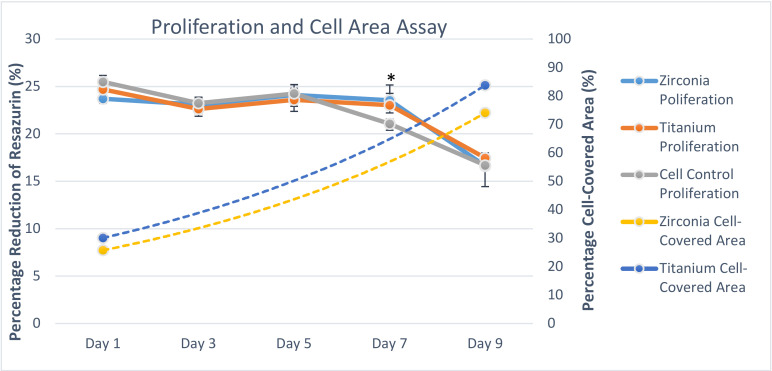
Cellular proliferation expressed as the average percentage of reduced resazurin in cells cultured on SBY-TZP and SBCpTi compared to a positive cell control. MC3T3 cells were seeded on to SBY-TZP (n = 8) and SBCpTi discs (n = 8) and a positive control (n = 8) and incubated for 1, 3, 5, 7, and 9 days. The proliferation assay determined the reduction of resazurin into resorufin and was measured at a wavelength of 570 nm with the subtraction of the 600 nm background using a microplate absorbance reader. A cell-covered area assay was included as a trendline. Cellular proliferation results are presented as mean ± standard deviation. * denotes a significant difference of the percentage reduction of resazurin of SBY-TZP and SBCpTi compared to the cell control (*p* < 0.05).

Results for cellular proliferation at 1, 3, 5, 7, and 9 days of incubation are shown in Figure 7. A 3 (substrate) × 5 (day) mixed factorial ANOVA was performed to examine the differences in cellular proliferation over time and across substrate (SBY-TZP, SBCpTi, or cell control). There was a significant main effect for time: F(4,24) = 207.76, *p* < 0.05. Post-hoc analyses indicated there was a significant difference in cellular proliferation between days 7 and all other days (*p* < 0.05), and day 9 and all other days (*p* < 0.05). There was no significant main effect for substrate, F(2,6) = 0.25, *p* > 0.05, nor interaction between time and substrate, F(2,6) = 0.63, *p* > 0.05. This indicates that cellular proliferation did not significantly differ across SBY-TZP or SBCpTi, nor did it differ based on time. The cell-covered area on the surfaces of the discs increased significantly across time (*p* < 0.05) but did not significantly differ across SBCpTi compared to SBY-TZP at any time point (Figure 7).

**Figure 8:**
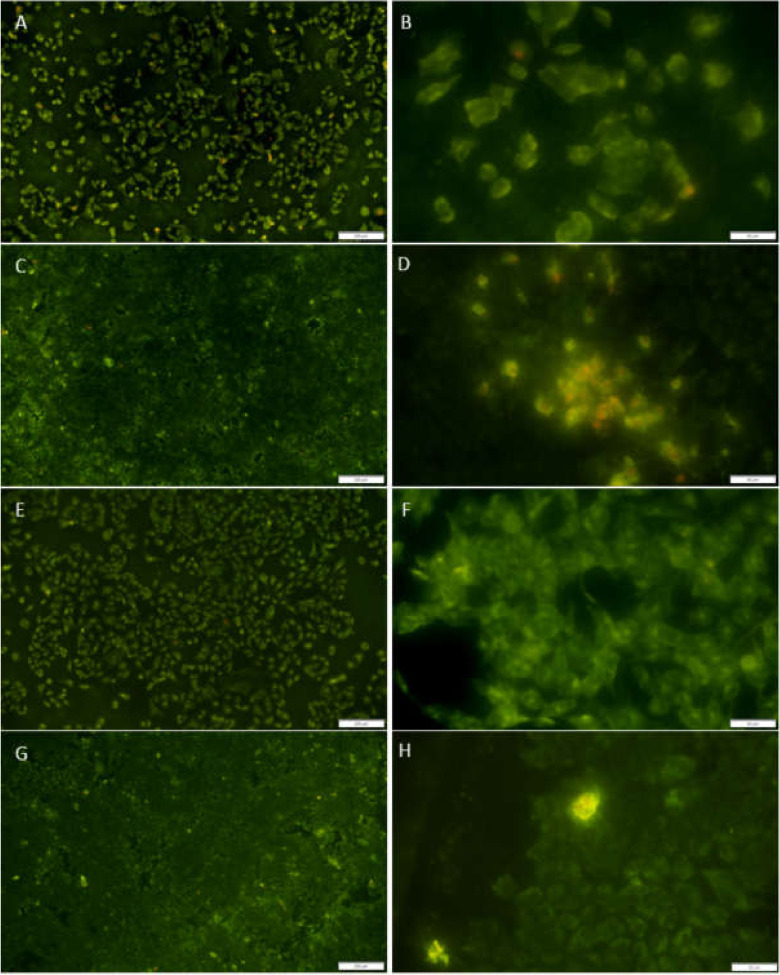

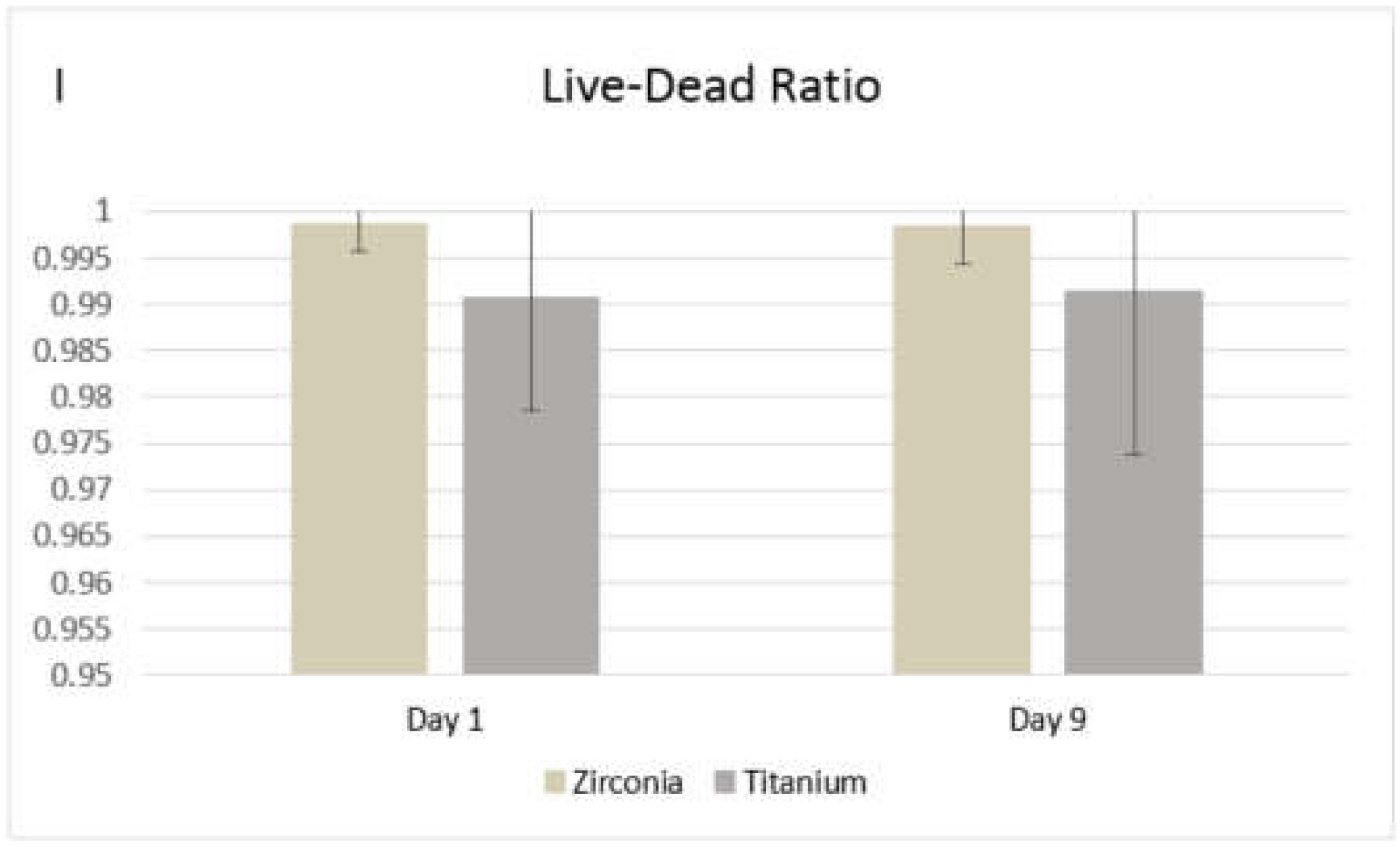
Cell viability of MC3T3 cells facilitated by SBCpTi and SBY-TZP surfaces. MC3T3 cells were seeded onto SBY-TZP (n = 4) and SBCpTi (n = 4) discs and incubated for 1 and 9 days. The cells were imaged with epifluorescence microscopy, a Cytopainter Cell Plasma Membrane Staining Kit (green), and propidium iodide (red). A–D Representative images of cellular viability across SBCpTi: day 1 and 10× magnification (**A**), day 1 and 40× magnification (**B**), day 9 and 10× magnification (**C**), day 9 and 40× magnification (**D**). E–H Representative images of cellular viability across SBY-TZP: day 1 and 10× magnification (**E**), day 1 and 40× magnification (**F**), day 9 and 10× magnification (**G**), day 9 and 40× magnification (**H**). The amount of fluorescence in a Cytopainter Cell Plasma Membrane Staining Kit (green) and propidium iodide (red) was measured to obtain a live-to-dead number ratio using ImageJ software. Results are presented as the median ± interquartile range (**I**).

The results for cell viability at 1 and 9 days of cultivation are shown in Figure 8. Day 1 showed a limited number of dead cells, with dominant live cell numbers for both SBCpTi and SBY-TZP. Day 9 showed a confluence of cells on the discs with a subsequent increase in dead cell numbers for both surfaces. A 2 (disc material) × 2 (day) mixed factorial ANOVA was performed to examine the differences in cellular viability (live–dead ratio) over time and across disc type (SBY-TZP or SBCpTi). There was no significant main effect for time, F(9, 20) = 0.01042, *p* > 0.99. However, a significant main effect for disc material was noted F(1, 20) = 10.45 *p* < 0.05. Post-hoc analyses indicated there was no significant difference in cellular viability between SBY-TZP and SBCpTi (*p* > 0.05). This suggests that cellular viability did not significantly differ across time or across disc material on both days 1 and 9.

#### 3.2.3. Cells Showed Improved Rates of Migration on Sandblasted Y-TZP Compared to CpTi Discs

**Figure 9:**
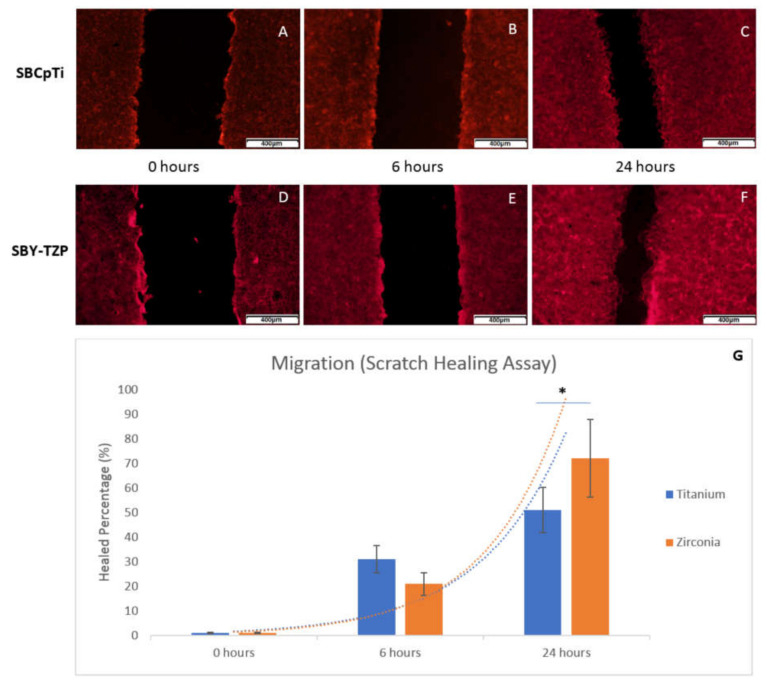
Migration assay results of MC3T3 cells across SBCpTi and SBY-TZP surfaces. MC3T3 cells were seeded onto SBY-TZP (n = 4) and SBCpTi (n = 4) discs and grown until confluent. Discs were scratched (two scratches per disc), and the migration of cells across the created gap was assessed at 0, 6, and 24 h post-scratch using Flash Phalloidin^TM^ Red solution and epifluorescence microscopy. (**A**–**C**) Representative images (10× magnification) of the migration of cells across SBCpTi at 0, 6, and 24 h, respectively. (**D**–**F**) Representative images (10× magnification) of the migration of cells across SBY-TZP at 0, 6, and 24 h, respectively. (**G**). Images were analysed, and the percentage of the area of the scratch healed at each time point was calculated using ImageJ software. Results are presented as median + interquartile range. * denotes a significant difference of percentage of the area covered on SBY-TZP discs compared with SBCpTi discs (*p* < 0.05).

The healing of the scratch area on each disc at 0, 6, and 24 h and healed percentage is evident in Figure 9. At 6 h, no significant difference in the percentage and subsequent rate of migration was evident for either surface (Figure 9B,E). After 24 h, zirconia had an average 72% healing rate of the scratched area (Figure 9F,G), which was significantly higher (*p* = 0.016) than the average 51% healing rate for titanium (Figure 9C). This showed that a significantly higher rate of migration of the cells was facilitated by the zirconia surface compared to the titanium surface.

## 4. Discussion

The aim of the present study was to characterise the zirconia implant surface and evaluate the behaviour of MC3T3-E1 cells on novel SBY-TZP and SBCpTi in vitro. The results showed improved viability, cytoskeletal arrangement, and attachment and migration of cells on the SBY-TZP surface compared with similarly modified titanium.

Previous studies have shown that a roughened zirconia surface will result in improved in vitro results compared to a machined or polished zirconia surface; therefore, the rationale of this investigation was to evaluate the osseointegration potential of a novel zirconia surface compared to a titanium surface [6,28,32,56,58,67]. In this study, micro- and nano-topographies were created on Y-TZP and CpTi surfaces by sandblasting and evaluated by laser scanning microscopy and SEM. Both Y-TZP and CpTi had a significant increase in surface topography measures following sandblasting, indicating that the subtractive treatment of sandblasting was successful for both CpTi and Y-TZP (Table 1).

The Sa value of 3.36 μm of Y-TZP in this study was relatively higher than other reported sandblasted zirconia ceramics with previously reported values ranging from Ra (arithmetical mean height of a line) or Sa values from 0.56 μm to 2.50 μm [29,41,44,55,58,68,69,70]. Several of these studies used a titanium surface for comparison that had roughness values higher than the sandblasted ceramic; whilst in this study, the roughness of the tested SBCpTi and SBY-TZP surfaces were very similar (Sa values of 3.41 μm and 3.36 μm respectively; no significant difference). However, it is evident in Figure 2 that whilst the Sa value and, therefore, the surface roughness of the two sandblasted surfaces are similar, the micro- and nano-topography of each surface is unique. The hybrid parameter Sdr value showed a significant increase in the surface area of the SBCpTi discs due to the development of a more prominent nano-topography (Table 1). Therefore, the SBCpTi surface was characterised by a higher concentration of nano-topographical features compared to SBY-TZP, despite having had the same surface treatment. This highlights the technical challenge of creating and optimising zirconia topography compared to titanium [18,19,20,42,43,44]. Our study confirmed these findings, showing titanium to have a far more consistently complex surface topography (Figure 3A,B) compared to the isolated areas of surface texture on the sandblasted zirconia surface (Figure 3C,D). This can be explained by the higher plasticity of titanium alloy compared to zirconia attributable to the inherent differences in toughness and brittleness of the bulk materials [44]. Sandblasting of the zirconia surface can trigger a tetragonal to monoclinic phase transformation, which is accompanied by a substantial increase in volume, subsequently inducing a compressive force at the surface. This force closes the fractured area, enhancing the resistance of the surface to further propagation. Further development of a nano-topography on the zirconia surface has been facilitated by subsequent acid-etching [6,48,56].

Despite the differing topography, SBCpTi and SBY-TZP had similar water contact angles of approximately 56 and 55 degrees respectively (Figure 4), classifying both surfaces as hydrophilic [38,61]. Although both SBCpTi and SBY-TZP had significant increases in Sa and Sdr and, a significant difference in surface wettability was observed between untreated titanium and SBCpTi whilst the surface wettability of SBY-TZP was not significantly different to untreated Y-TZP (Figure 4E). Interestingly, there was no significant difference in Sa or Sdr values between untreated Y-TZP and CpTi surfaces, although there was a significant difference in the surface wettability. Consequently, it is apparent that factors other than surface roughness may play a significant role in increasing the hydrophilicity of a surface as well as influencing cellular interaction. The interactions of cells and tissues with foreign materials are governed not only by the physical properties, such as roughness and topography, but also the chemical properties of the material surface such as hydrophilicity [30,33,61,71,72].

It was apparent that the differing sandblasted topographies of titanium and zirconia facilitated differing MC3T3-E1 cell morphologies (Figure 5,Figure 6). After 24 h of incubation, there was a marked increase in the number of cell-to-cell contacts and well-organised cellular extensions on SBY-TZP compared with SBCpTi, indicating improved cellular adhesion on SBY-TZP [73]. This was confirmed with the quantitative analysis of actin fluorescence that was higher (statistically significant) in zirconia discs compared to titanium discs (Figure 5). This was supported by the findings of the viability assay (Figure 8) that showed a greater number of cellular contacts on zirconia compared to titanium. In contrast, Yamashita et al. compared sandblasted zirconia and titanium surfaces, with comparable surface roughness (Ra 1.01 and 1.03 μm respectively), and found no significant difference in cell attachment or morphology [58]. Han et al. used the same cell line, MC3T3-E1, to compare titanium and zirconia surfaces with similar roughness and also found no significant difference in cell morphology; however, Bergemann et al. found that the surface roughness Ra values from 1.22 to 1.32 µm resulted in reduced cell spreading with shortened actin filaments on zirconia [55,73]. Additionally, Strickstrock et al. tested two sandblasted Y-TZP surfaces of Sa values of 1.01 μm and 2.50 μm, with the more roughened surface not supporting cell adhesion as efficiently [44]. These studies highlight not only the importance of surface characteristics but also the optimisation of these surfaces to obtain ideal cellular interaction and long-term success. In this study, higher roughness values with a Sa value of 3.36 μm resulted in zirconia having improved cellular spreading. This may also have facilitated the significantly increased rate of migration (Figure 9), as cells were able to produce a greater number and length of cellular extensions and filopodiae. Further research into different surface topographies in combination with the chemical state of the implant material surface is important to allow for the complete optimisation of the osseointegration potential of this novel zirconia surface. Chemical properties such as hydrophilic status and charge may have a direct impact on the initial adsorption of proteins and subsequently promote better cell adhesion and spread [33,34,73]. Optimal cell adhesion is mediated by the absorption of cell adhesion-mediating molecules (such as fibronectin and vitronectin), which then makes the surface accessible to cell adhesion receptors [71,72]. Therefore, the improved cell morphology and migration of the MC3T3-E1 cells on the SBY-TZP surface compared to SBCpTi may relate to the differences in the chemical properties of the two surfaces. Future studies of this novel zirconia material will examine the role of its chemical properties in interactions with cells.

No significant difference in cellular proliferation was evident between the SBCpTi and SBY-TZP surfaces (Figure 7). At day 9, cell proliferation began to decline, which was likely a result of contact inhibition as cells reached confluence. Additionally, the higher cell numbers would exhaust nutrients in the media and create an associated build-up of toxic lactic acid, which may result in an increase in cellular death, correlating to the findings of the cell-covered area on day 9 [17,74]. This was also confirmed by a decrease in the live–dead ratio, with increasing dead cell numbers. However, cellular viability was not statistically significant on SBY-TZP compared to SBCpTi on days 1 and 9, although titanium did not show any cytotoxic effects. Similarly, other studies reported no difference in cellular proliferation between the tested titanium (Ra of 1.04 to 1.43 μm) and zirconia surfaces (Ra of 0.93 to 1.41 μm) [48,59,70]. Strickstrock et al. also found that zirconia and titanium surfaces of similar surface roughness showed no significant difference in cellular proliferation or viability; however, a more roughened zirconia surface (Sa of 2.50 μm) did show a more pronounced reduction in cell density and proliferation of primary human osteoblasts [44]. This further highlights the importance of surface optimisation to cater for ideal cellular responses [44,48,59,70]. In this study, no significant difference was seen in viability and proliferation, indicating that long-term osseointegration is reliant on other properties than just surface topography.

This study showed that the cell-stimulating properties of zirconia were comparable or exceeded that of titanium. Sandblasting resulted in higher roughness values with a Sa value of 3.36 μm giving zirconia a greater osseointegration potential due to its significantly increased viability, cellular spreading, and rate of migration compared to titanium. However, this is within the limitations of an in vitro study. Further in vitro and in vivo animal studies would be necessary to further assess this novel zirconia surface as a suitable material for dental implants and an alternative to titanium-based dental implants. Further study of the physiochemical changes of the surfaces would be important for optimisation to determine if the improved cellular interactions were due to chemical or topographical changes, or, most likely, a combination of both.

## 5. Conclusions

Modifying the surface roughness of zirconia and titanium discs with sandblasting resulted in similar surface roughness measures for both zirconia and titanium, although zirconia failed to achieve a nano-topography similar to that seen in titanium with a significantly higher surface area. Despite this, within the limitations of an in vitro study, sandblasted yttria partially stabilised zirconia was noted to enhance viability, migration, and spreading when compared to titanium. However, further research is needed to characterise the chemical properties and subsequent effects on the cellular response of this novel zirconia surface. Additionally, the negative effect of sandblasting zirconia surfaces on the mechanical properties should be evaluated further. Collectively, this study confirms the biocompatible nature of this novel zirconia surface and its potential for the application as a dental implant owing to improved cellular response.

## Figures and Tables

**Table 1:** Results of the topographical analyses by laser scanning microscope on untreated and sandblasted Yttria-tetragonal zirconia polycrystal (Y-TZP) and titanium (CpTi). Data are presented as mean ± standard deviation n = 3 sites per discs (into 3 discs). Sa, arithmetic mean height; Sdr, developed interfacial area ratio; Ssk, skewness; Sku, kurtosis; Str, texture aspect ratio. * indicates significant difference (*p* < 0.001) between untreated surfaces. # indicates a significant difference (*p* < 0.001) between sandblasted and untreated surfaces. + indicates a significant difference (*p* < 0.001) between sandblasted surfaces.

Material	Surface	Sa (µm)	Sdr (Units)	Ssk (Units)	Sku (Units)	Str (Units)
Y-TZP	Sandblasted	3.36 ± 0.49 ^#^	3.3 ± 0.1 ^#+^	0.9 ± 0.2 ^#^	8.1 ± 1.4	1.1 ± 0.2 ^#^
	Untreated	1.69 ± 0.20 ^#^	1.3 ± 0.01 ^#^	−0.6 ± 0.8 ^#*^	5.0 ± 2.6	0.02 ± 0.01 ^#*^
CpTi	Sandblasted	3.41 ± 0.51 ^#^	4.3 ± 0.02 ^#+^	0.4 ± 0.6 *	8.7 ± 1.5 ^#^	0.8 ± 0.05
	Untreated	1.51 ± 0.63 ^#^	1.2 ± 0.17 ^#^	0.1 ± 0.3	4.2 ± 0.9 ^#^	0.7 ± 0.4 *

## Abbreviations

Y-TZP (Yttria-tetragonal zirconia polycrystal); SBY-TZP (Sandblasted yttria-tetragonal zirconia polycrystal); CpTi (Commercially pure titanium); SBCpTi (Sandblasted commercially pure titanium).
